# The Impact of Flap Creation Methods for Sub-Bowman’s Keratomileusis (SBK) on the Central Thickness of Bowman’s Layer

**DOI:** 10.1371/journal.pone.0124996

**Published:** 2015-05-04

**Authors:** Zhe Xu, Meixiao Shen, Liang Hu, Xiran Zhuang, Mei Peng, Di Hu, Jing Liu, Jianhua Wang, Jia Qu, Fan Lu

**Affiliations:** 1 School of Ophthalmology and Optometry, Wenzhou Medical University, Wenzhou, Zhejiang, China; 2 Department of Ophthalmology, Bascom Palmer Eye Institute, University of Miami, Miami, FL, United States of America; Medical University of South Carolina, UNITED STATES

## Abstract

**Purpose:**

To determine the impact of flap creation methods for sub-Bowman’s keratomileusis (SBK) on central Bowman’s layer thickness.

**Methods:**

SBK flaps were made by Moria microkeratome for 20 subjects and by femtosecond (FEMTO) laser for 21 subjects. Corneal sublayer thicknesses were measured by ultra-high resolution optical coherence tomography before SBK and at 1 day, 1 week, 2 weeks, and 1 month afterwards. Each subject was imaged twice on each visit. Thicknesses of central epithelium, Bowman’s layer, flap, and total cornea were calculated using a custom-made automated image processing algorithm. The repeatability of sublayer thickness measurements was tested by the intraclass correlation coefficient (ICC) and by the coefficient of repeatability (CoR) at 1 week post-SBK.

**Results:**

ICCs of the Moria and FEMTO groups were ≥0.959 and ≥0.961 respectively for all sublayer measurements. The segmentation CoRs were less than 6.78% and 5.63% respectively. For both groups, microdistortions were present in the epithelium and Bowman’s layer after SKB. The flap thickness of the Moria group was 9.8 μm (95% confidence interval: 4.8 – 14.8μm) thinner than the FEMTO group one day after SBK (independent samples t-test, P < 0.05). Bowman’s layer became thicker by 1.6 ± 1.1 μm and 1.7 ± 1.6 μm one day post-SBK for the Moria and FEMTO groups (repeated ANOVA, P < 0.05) and then remained stable. Corneal and sublayer thickness were similar between the two groups.

**Conclusions:**

Central Bowman’s layer thickness increased 1 day post-SBK. Flap creation by Moria microkeratome and femtosecond laser did not have significantly different impacts on Bowman’s layer thickness following SBK.

**Trial Registration:**

Chinese Clinical Trial Registry (ChiCTR) NO: ChiCTR-OCH-14004525

## Introduction

Bowman′s layer is an acellular and non-regenerating layer positioned between the basement membrane of the corneal epithelium and the anterior stroma [1,2]. In human corneas, the thickness of Bowman′s layer is approximately 8 to 20 μm [3,4]. Unmyelinated nerve axons irregularly penetrate Bowman′s layer to support epithelial innervation [5]. Structural and functional disorders of Bowman′s layer, like that caused by subepithelial fibrous pannus [6], may lead to corneal diseases, such as keratoconus, Fuch′s corneal dystrophy, and Thiel-Behnke dystrophy [4,7–9].

Laser in situ keratomileusis (LASIK) is the most popular refractive surgery technique for myopia patients to correct refractive errors. Corneal flap creation, one of the critical procedures during LASIK, helps to maintain corneal structures such as Bowman′s layer and the epithelium [10]. Sub-Bowman′s keratomileusis (SBK) was developed from LASIK by using a mechanical microkeratome or femtosecond laser to create a thinner corneal flap of 90 to 110 μm [11]. The evolution of SBK with the thin flap has improved the efficacy, tolerability, and safety of refractive surgery for myopia [11]. Despite the improvements in refractive surgery with SBK, potential complications, such as flap folds, flap buttonhole, and post-surgery keratectasia, are still significant threats to the patient′s vision [12]. Therefore, monitoring and maintaining the health of corneal tissue after surgery is still one of the most important issues for clinical ophthalmologists and researchers. Previous studies have investigated the changes in corneal curvature [13], epithelial thickness [14–17], and corneal flap thickness [12,17,18] after refractive surgery. Some researchers reported that the method of flap creation influenced these corneal structural changes [11–13]. However, little has been published regarding the impact of methods for creation of thin flaps by SBK on the thickness changes of Bowman′s layer.

Several methods, such as very high-frequency digital ultrasound [12] and in vivo confocal microscopy [19], can measure the thicknesses of the corneal epithelium and Bowman′s layer. However, those methods were not precise enough to investigate the micro-structural changes of cornea. With a custom-built, ultra-high resolution optical coherence tomography (UHR-OCT) instrument that has an axial resolution of 3 μm, the corneal epithelium and Bowman′s layer can be exactly imaged by without physical contact and non-invasively [3,20–22]. The goal of this study was to determine by UHR-OCT the impact of SBK flap creation by microkeratome and femtosecond laser on the thickness of Bowman′s layer.

## Methods

### Subjects

This study was approved by the Office of Research Ethics, Wenzhou Medical University, Wenzhou, Zhejiang, China. All subjects were recruited at the Refractive Surgery Division of the Affiliated Eye Hospital of Wenzhou Medical University. Written consent was obtained from each subject, and all subjects were treated according to tenets of the Declaration of Helsinki. As required for conducting a clinical trial, this study was registered in the Chinese Clinical Trial Registry (ChiCTR NO. ChiCTR-OCH-14004525, registry URL: http://www.chictr.org). The protocol for this trial and CONSORT checklist are available as S1 Checklist and S1 Protocol.

**Fig 1:**
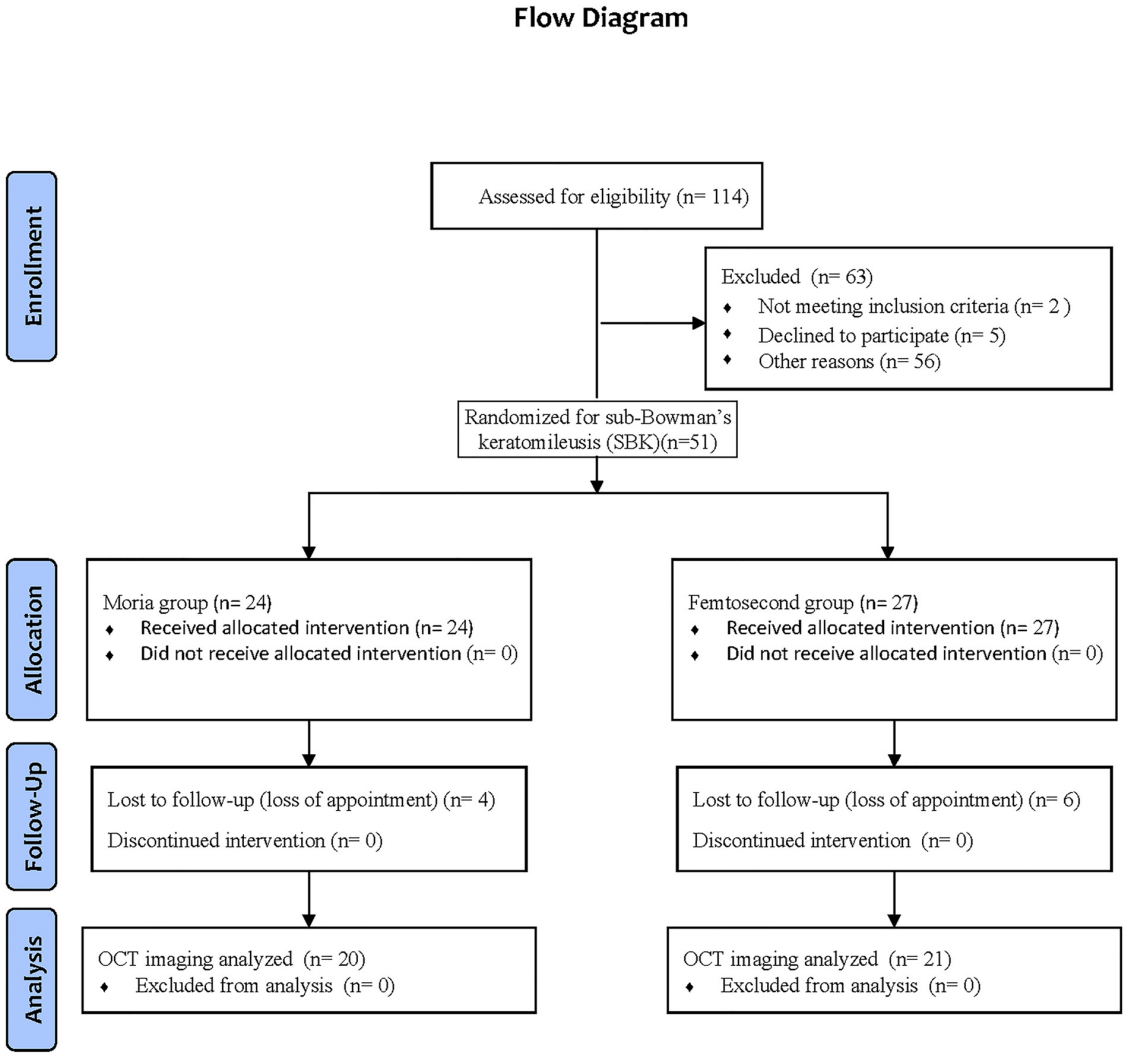
CONSORT flow diagram of OCT imaging observations on flap creation methods for sub-Bowman′s keratomileusis (SBK).

Inclusion criteria consisted of no history of ocular pathologic conditions, trauma, previous ocular surgery, or recent ocular medication use. The refractive error before surgery was between -1.00 to -8.00 diopters (D) of myopia with ≤ -2.00 D of refractive astigmatism and stable for 1 year. The best spectacle-corrected visual acuity (BSCVA) was more than 20/20 in each eye. Patients wearing soft contact lenses were required to stop wearing them 2 weeks before surgery, and patients wearing rigid gas permeable lenses were required to stop wearing them 1 month before surgery. Patients were evaluated before surgery and 1 day, 1 week, 2 weeks, and 1 month after surgery. All the clinical examinations included measurements of manifest refraction, uncorrected visual acuity (UCVA), BSCVA, slit-lamp biomicroscopy, and corneal topography. Only subjects who completed the pre-operative visit and all 4 post-operative visits were included in the data analysis (Fig 1).

For one group, designated the Moria group (13 men, 7 women; mean age ± standard deviation age 25.2±4.1 years), the corneal flaps were created by the One-Use Plus SBK Moria microkeratome (Moria, Antony, France). For the other group, designated the FEMTO group (13 men, 8 women; 24.2±4.0 years), the corneal flaps were created with the IntraLase Femtosecond Laser (Abbott Medical Optics, Santa Ana, CA, USA). The intended flap thickness for both groups was 110 μm. The ablations were performed by a 400-Hz Mel-80 excimer laser (Carl Zeiss Meditec, Oberkochen, Germany). One surgeon (LH) conducted all of the surgical procedures. No patients had severe complications after SBK surgery.

### Instruments

The detail parameters of our custom-built UHR-OCT system have been described in previously published papers [3,20–22]. It uses a superluminescent diode light source with a broad bandwidth of 100 nm centered at wavelength of 840 nm, which achieves approximately 3-μm axial resolution in corneal tissue. The acquisition rate was 24k A-lines per second. Each B-scan image consisted of 1,365 pixels per 2.01 mm for scan depth and 2,048 pixels per 8.41 mm for scan width. One eye of each patient was randomly included using Microsoft Excel′s (Microsoft Crop, Redmond, WA, USA) random number generator. The horizontal scan pattern was performed twice by one operator (ZX) before surgery and at 1 day, 1 week, 2 weeks, and 1 month after SBK. Each subject was asked to fixate on a visual target positioned in front of the contralateral eye while the test eye was being scanned. During the interval between scans, the subject was required to sit back and rest for 15 minutes prior to repositioning the chin.

**Fig 2:**
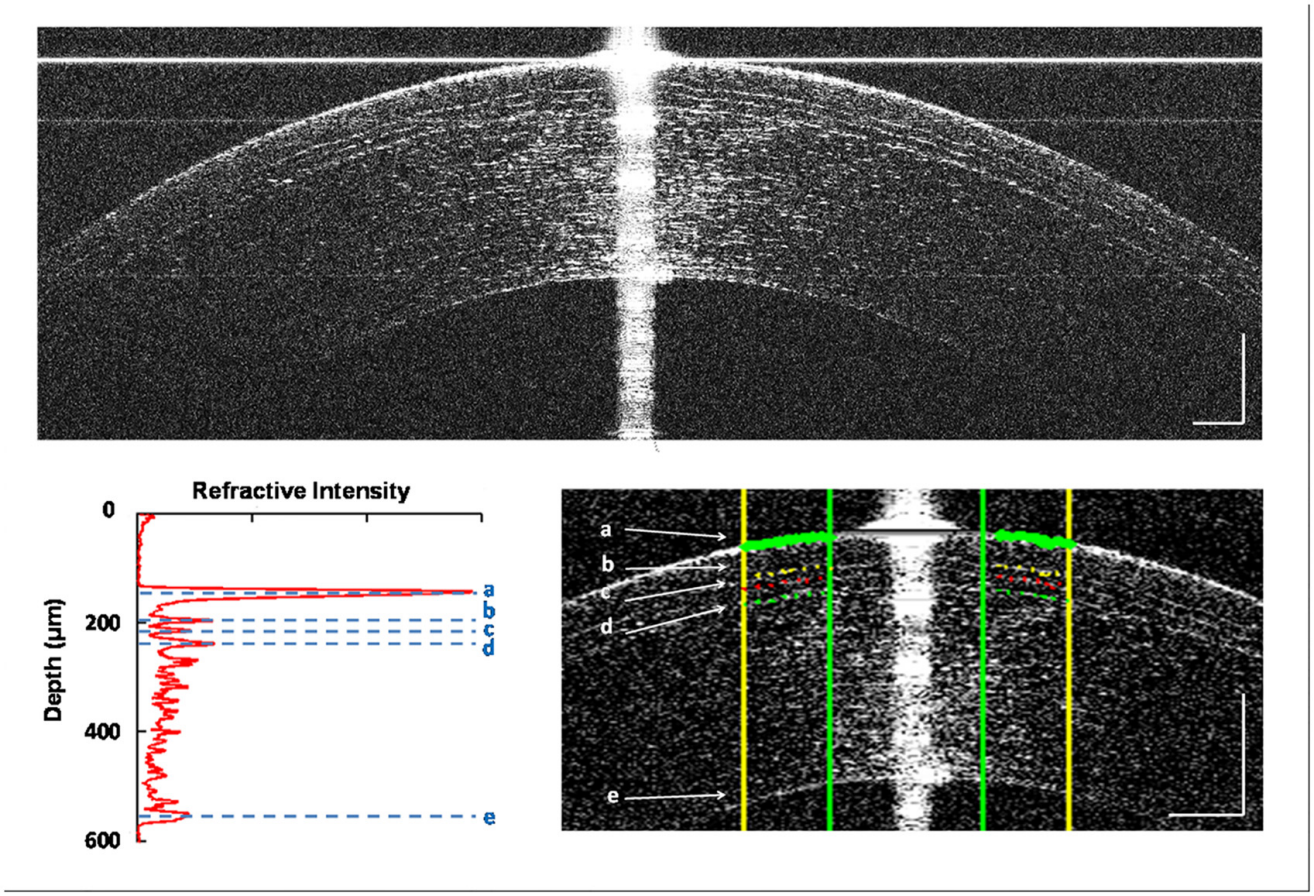
UHR-OCT image illustration of the image processing procedure. (A) Original UHR-OCT image taken 1 day after SBK for which the flap was made with the One-Use Plus SBK Moria microkeratome. Similar images were obtained after SBK in which the flap was made by FEMTO laser. (B) Longitudinal reflective profile of the detected central cornea sublayers. (C) Automated segmentation of central corneal region virtually eliminated the vertical specular reflex and horizontal artifacts. The distances between (a) and (b), (b) and (c), (a) and (d), (a) and (e) represent the thickness of the epithelium, Bowman′s layer, corneal flap, and total cornea thickness respectively. Bars = 250 μm.

The automated image processing algorithm developed in Matlab-based software (MathWorks, Inc., Natick, MA, USA) for sublayer segmentation was reported in previous papers [21,22]. First, the algorithm reduced the background noise and filtered out the artifacts of horizontal and specular reflections from the original images (Fig 2). The central 128 axial scans of 0.5-mm width were removed. Second, the central region was defined as the first peak of the air-epithelium interface. The 0.5-mm regions around both sides of the removed hyperreflective reflex were processed to create a longitudinal profile. Along each axial scan, the first, second, and third peaks corresponded to the air-epithelium, epithelium-Bowman′s layer, and Bowman′s layer-stroma interfaces (Fig 2). For post-SBK subjects, a forth peak was present that indicated the flap interfaces. The final peak was the endothelium-aqueous interface. The epithelium and Bowman′s layer thicknesses were defined as the distances between the adjacent two peaks. Corneal flap thickness was determined as the distance from the first to the forth peak. The distance between the first and final peak represented the corneal thickness. A refractive index of 1.376 was used to calculate thickness results from optical distances. If the peak locations did not match the sublayer boundaries on the UHR-OCT images, manual segmentations were performed. The average of two measurements was taken as the thickness for each sublayer.

### Statistical analysis

The mean values and standard deviations (SDs) were calculated for thicknesses of the epithelium, Bowman′s layer, flap, and total cornea. Data analysis was performed by the Statistical Package for the Social Sciences software (ver. 17, SPSS Inc., Chicago, IL, USA). The intrasession repeatability of two UHR-OCT measurements 1 week after surgery was calculated as the intraclass correlation coefficient (ICC) and coefficient of repeatability (CoR). ICC was used to evaluate the proportion of variability between two measurement differences compared to overall variability. CoR was defined as two SDs of the difference between two measurements of the same subject by one operator. CoR% was defined as the CoR value divided by overall mean and then multiplied by 100 [24]. The agreement of the two measurements was evaluated by 95% limit of agreement (LoA) using the Bland and Altman method. The 95% LoA was defined as the mean value of the difference ± 1.96 SD of the difference [25]. Repeated-measures analysis of variance (ANOVA) and post hoc testing were used to test the sublayer changes over the pre- and post-operative visits. Independent samples t-test was used to make the comparison between the two flap formation methods. 95% confidence intervals (CIs) were used to calculate the thickness differences between Moria and FEMTO groups. P < 0.05 was defined as statistically significant.

The sample size was estimated by G*Power (version 3.1.9) using t test analysis with 5% type I error, 95% statistical power and effective size of 1.0. The minimum sample size calculated by Bowman′s layer changes was 13 for both Moria and FEMTO groups [23]. In the current study, sample sizes of 20 subjects for both Moria group and 21 subjects for FEMTO group were more than adequate.

## Results

**Fig 3:**
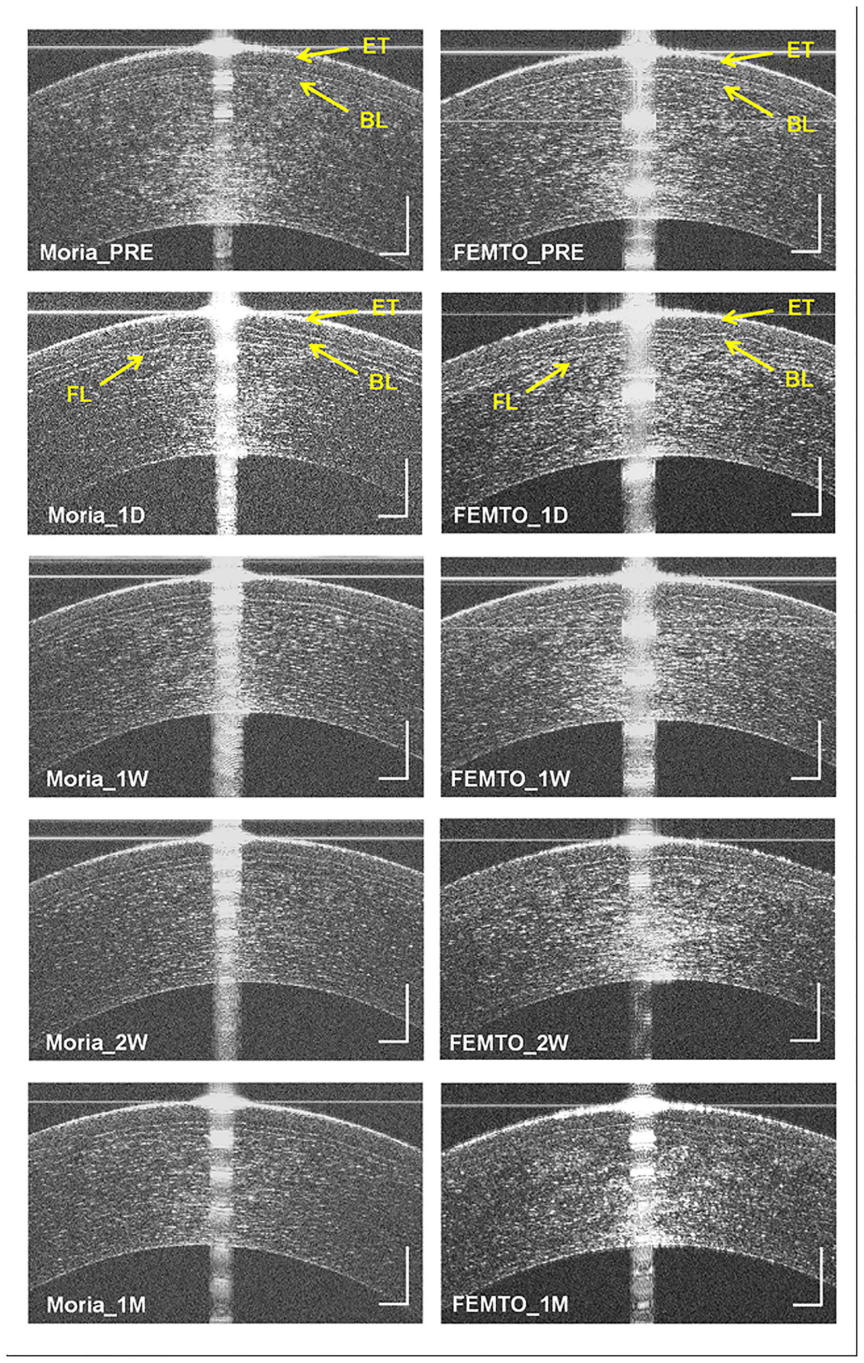
UHR-OCT images taken before and after sub-Bowman′s keratomileusis (SBK) surgery in the Moria and FEMTO groups. (A) UHR-OCT image before Moria SBK. (B) UHR-OCT image of 1 day post Moria SBK. (C) UHR-OCT image of 1 week post Moria SBK. (D) UHR-OCT image of 2 weeks post Moria SBK. (E) UHR-OCT image of 1 month post Moria SBK. (F) UHR-OCT image before FEMTO SBK. (G) UHR-OCT image of 1 day post FEMTO SBK. (H) UHR-OCT image of 1 week post FEMTO SBK. (I) UHR-OCT image of 2 weeks post FEMTO SBK. (J) UHR-OCT image of 1 month post FEMTO SBK. ET, epithelium; BL, Bowman′s layer; FL, flap. Bars = 250 μm.

The spherical equivalent (SE) refractions before surgery of the Moria and FEMTO groups were -5.24±1.84 D and -4.73±1.68 D respectively (independent sample t-test, P > 0.05). At the 1 month follow-up visit after surgery, SE refractions for the Moria and FEMTO groups were 0.04±0.36 D and 0.15±0.29 D respectively (independent sample t-test, P > 0.05). All the eyes had UCVA of 20/20 or better. Microdistortions in the epithelium, Bowman′s layer, and flap were imaged 1 day after SBK in both groups (Fig 3). At 1 month post-SBK, the microdistortions in all layers still existed in UHR-OCT images (Fig 3).

The repeatability of the automated image processing algorithm was calculated from UHR-OCT images taken at 1 week after surgery. The ICCs for segmentation of all corneal layers (Table 1) were ≥ 0.959 for the Moria group and ≥ 0.961 for the FEMTO group. The CoR% values were less than 6.78% and 5.63% of the segmentations for each of the corneal layers of Moria and FEMTO groups respectively. The 95% LoA for the segmentation of Bowman′s layer for FEMTO group was -0.97 to 0.92 μm, which was narrower than that for the Moria group, -1.15 to 1.25 μm.

**Table 1:** Repeatability of automated UHR-OCT image processing algorithm for the Moria and FEMTO groups.

	M1 (μm)	M2 (μm)	Dif (μm)	ICC	CoR (μm)	CoR (%)	95% LoA (μm)
**Moria group (*n* = 20)**							
** Epithelium**	**54.4 ± 3.6**	**54.9 ± 3.9**	**0.51 ± 1.22**	**0.973**	**2.45**	**4.47**	**-1.89 to 2.90**
** Bowman's layer**	**18.0 ± 1.5**	**18.1 ± 1.6**	**0.05 ± 0.61**	**0.959**	**1.23**	**6.78**	**-1.15 to 1.25**
** Flap**	**95.5 ± 7.8**	**95.5 ± 7.5**	**0.05 ± 1.80**	**0.986**	**3.59**	**3.76**	**-3.47 to 3.57**
** Total cornea**	**431.6 ± 23.6**	**430.5 ± 24.2**	**-1.04 ± 4.29**	**0.992**	**8.57**	**1.99**	**-9.44 to 7.36**
**FEMTO group (*n* = 21)**							
** Epithelium**	**53.2 ± 3.1**	**53.1 ± 2.9**	**-0.05 ± 1.02**	**0.970**	**2.05**	**3.85**	**-2.06 to 1.96**
** Bowman's layer**	**17.2 ± 1.2**	**17.2 ± 1.3**	**-0.03 ± 0.48**	**0.961**	**0.97**	**5.63**	**-0.97 to 0.92**
** Flap**	**106.3 ± 8.6**	**106.2 ± 8.2**	**-0.07 ± 2.00**	**0.986**	**4.00**	**3.76**	**-3.99 to 3.85**
** Total cornea**	**443.7 ± 37.8**	**442.5 ± 38.8**	**-1.16 ± 4.71**	**0.996**	**9.42**	**2.13**	**-10.40 to 8.07**

M1, first thickness measurement at post-operative week 1; M2, second thickness measurement at post-operative week 1; Dif, the difference between M1 and M2; ICC, intraclass correlation coefficient; CoR, coefficient of repeatability; 95% LoA, 95% limit of agreement.

**Fig 4:**
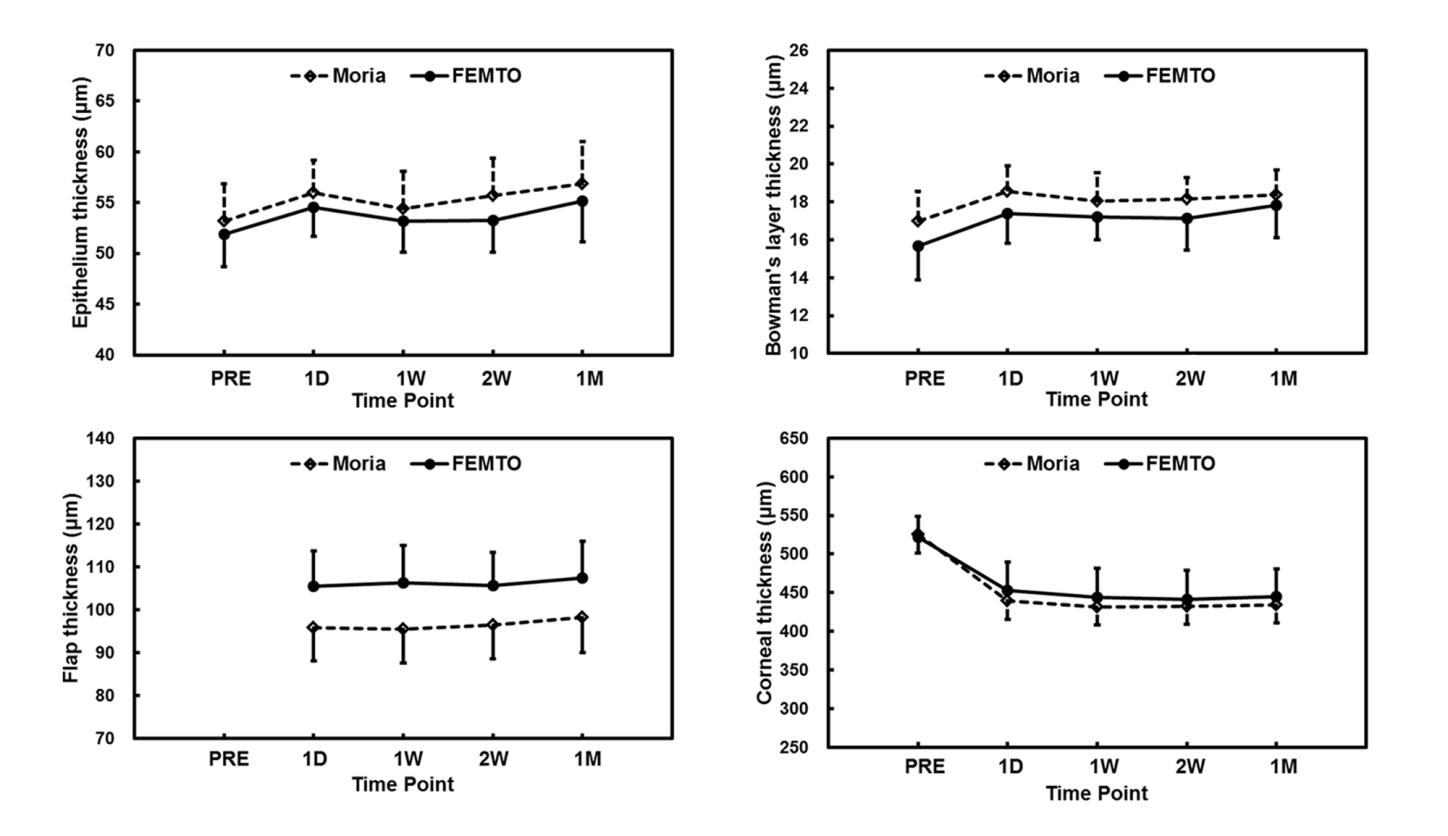
Thickness changes in the corneal sublayers of the Moria and FEMTO groups. (A) Epithelial thicknesses measured by UHR-OCT in both groups were increased sharply at 1 day after SBK, and then continued to increase to the end of the follow-up period at 1 month. (B) Bowman′s layer thicknesses also increased by 1 day after SBK, but then remained steady afterwards. (C). Changes in flap thicknesses for both groups were not significantly until the second week post-SBK. Between two weeks and one month, the increase in flap thickness was small but significant for both groups. (D) Total corneal thickness for both groups decreased sharply by 1 day after SBK. Though the decreases were small between 1 day and 1 week after SBK, they were significant. Small but significant decreases continued from 2 weeks through the end of the follow-up period. PRE, pre-SBK; D, day post-SBK; W, week post-SBK; M, month post-SBK.

Compared to the pre-SBK epithelium thickness, one day after SBK the thickness increased significantly by 2.8 ± 3.2 μm and 2.6 ± 1.7 μm for the Moria and FEMTO groups respectively (repeated ANOVA, P < 0.05, Tables 2 and 3, Fig 4). The epithelial thickness continued to increase by 1.2 ± 1.6 μm and 2.0 ± 3.3 μm between 2 weeks and 1 month after Moria and FEMTO surgery respectively (repeated ANOVA, P < 0.05).

**Table 2:** UHR-OCT measurements of central corneal sublayer thicknesses before and after sub-Bowman′s keratomileusis (SBK).

	PRE	1D	1W	2W	1M
**Moria group (*n* = 20)**					
** Epithelium (μm)**	**53.1 ± 3.7**	**56.0 ± 3.2** *	**54.4 ± 3.6** *	**55.7 ± 3.7** *	**56.9 ± 4.1** *
** Bowman's layer (μm)**	**17.0 ± 1.6**	**18.5 ± 1.4** *	**18.0 ± 1.5**	**18.1 ± 1.1**	**18.4 ± 1.3**
** Flap (μm)**	-	**95.8 ± 7.6**	**95.5 ± 7.8**	**96.5 ± 7.9**	**98.2 ± 8.2** *
** Total cornea (μm)**	**525.8 ± 25.0**	**439.5 ± 24.0** *	**431.6 ± 23.6** *	**432.0 ± 23.1**	**434.2 ± 23.0** *
**FEMTO group (*n* = 21)**					
** Epithelium (μm)**	**51.9 ± 3.2**	**54.5 ± 2.8** *	**53.2 ± 3.1** *	**53.2 ± 3.1**	**55.2 ± 4.0** *
** Bowman's layer (μm)**	**15.7 ± 1.8**	**17.4 ± 1.5** *	**17.2 ± 1.2**	**17.1 ± 1.7**	**17.8 ± 1.7**
** Flap (μm)**	-	**105.6 ± 8.2**	**106.3 ± 8.6**	**105.7 ± 7.6**	**107.5 ± 8.5** *
** Total cornea (μm)**	**521.7 ± 27.2**	**453.1 ± 36.8** *	**443.7 ± 37.8** *	**441.7 ± 237.4**	**445.0 ± 36.0** *

PRE, before SBK; 1D, 1 day post-SBK; 1W, 1 week post-SBK; 2W, 2 weeks post-SBK; 1M, 1 month post-SBK.

*P < 0.05 compared with the preceding visit.

**Table 3:** UHR-OCT measured changes of central corneal sublayer thicknesses between visits after sub-Bowman′s keratomileusis (SBK).

	1D—PRE	1W—1D	2W—1W	1M—2W
**Moria group (*n* = 20)**				
** Epithelium (μm)**	**2.8 ± 3.2** *	**-1.6 ± 2.6** *	**1.3 ± 2.6** *	**1.2 ± 1.6** *
** Bowman's layer (μm)**	**1.6 ± 1.1** *	**-0.5 ± 1.8**	**0.1 ± 1.3**	**0.2 ± 1.0**
** Flap (μm)**	-	**-0.3 ± 2.4**	**1.0 ± 2.8**	**1.7 ± 2.3** *
** Total cornea (μm)**	**-86.4 ± 26.7** *	**-7.9 ± 6.1** *	**0.5 ± 3.7**	**2.2 ± 2.2** *
**FEMTO group (*n* = 21)**				
** Epithelium (μm)**	**2.6 ± 1.7** *	**-1.3 ± 2.7** *	**0.0 ± 2.7**	**2.0 ± 3.3** *
** Bowman's layer (μm)**	**1.7 ± 1.6** *	**-0.2 ± 1.4**	**-0.1 ± 1.3**	**0.7 ± 1.2**
** Flap (μm)**	-	**0.7 ± 3.2**	**-0.6 ± 3.1**	**1.8 ± 3.3** *
** Total cornea (μm)**	**-68.6 ± 26.9** *	**-9.5 ± 9.7** *	**-2.0 ± 8.5**	**3.3 ± 8.3** *

PRE, before SBK; 1D, 1 day post-SBK; 1W, 1 week post-SBK; 2W, 2 weeks post-SBK; 1M, 1 month post-SBK.

*P < 0.05 compared with the preceding change in thickness.

The thickness of Bowman′s layer increased by 1.6 ± 1.1 μm in the Moria group and 1.7 ± 1.6 μm in the FEMTO group one day after SBK (repeated ANOVA, P < 0.05, Tables 2 and 3, Fig 4). However, changes in Bowman′s layer thickness between 1 day and 1 month after SBK were not significant for either flap creation method.

At each time period after SBK, flap thickness in the Moria group was significantly thinner than that in the FEMTO group (independent samples t-test, P < 0.05, Table 2). On the first day after SBK, the flap thickness of the Moria group was 9.8 μm (95% CI: 4.8–14.8μm) thinner than the FEMTO group (independent samples t-test, P < 0.05). During the follow-up period, only between 2 weeks and 1 month did the flap thicknesses increase significantly for the two groups (repeated ANOVA, P < 0.05, Table 3, Fig 4).

After excimer laser ablation, the total thickness of the central cornea at one day after SBK decreased by 86.4 ± 26.7 μm and 68.6 ± 26.9 μm for the Moria and FEMTO groups respectively (repeated ANOVA, P < 0.05, Table 3, Fig 4). The thickness continued to decrease by 7.9 ± 6.1 μm in the Moria group and 9.5 ± 9.7 μm in the FEMTO group between 1 day and 1 week after SBK (repeated ANOVA, P < 0.05, Table 3, Fig 4). Between 2 weeks and 1 month after SBK, the central cornea thicknesses increased by 2.2 ± 2.2 μm for the Moria group and 3.3 ± 8.3 μm in the FEMTO group (repeated ANOVA, P < 0.05, Table 3, Fig 4).

## Discussion

High resolution images of Bowman′s layer may help better understand the impact of different refractive surgery procedures. Distinct images of Bowman′s layer have been illustrated in previous studies. Using UHR-OCT, Tao et al. reported that the central Bowman′s layer thickness was nearly 18 μm for healthy subjects [3]. Schmoll et al., using a complementary metal-oxide-semiconductor-based OCT instrument with an axial resolution of 1.3 μm in corneal tissue, reported a similar value for Bowman′s layer, 19 μm, in normal subjects [26]. After photorefractive keratectomy (PRK), Schmoll et al. found that Bowman′s layer was degenerated and the thickness varied from 10 to 18 μm. Germundsson et al., using in vivo confocal microscopy, reported Bowman′s layer of about 14 μm [27]. In a recently published paper by Yao et al., microdistortions in Bowman′s layer after femtosecond laser-assisted LASIK and small incision lenticule extraction were captured by a commercial SD-OCT instrument [28]. The number of twisted Bowman′s layer segments was defined to quantify the microdistortions. The number of microdistortions decreased at 1 week and then remained stable. In addition, histopathological reports by Sykakis et al. found Bowman′s layer breaks in the keratoconus patients [29]. Therefore, in vivo study of Bowman′s layer structural characteristics and thickness is critical to understand the role in corneal tissue and related visual quality issues.

To our knowledge, the current study is the first study of Bowman′s layer thickness after SBK surgery. We found that the thickness of Bowman′s layer significantly increased by 2 μm one day after SBK in both the Moria and FEMTO groups. The thickness remained stable between 1 day and 1 month post-SBK. Moreover, microdistortions in Bowman′s layer and in the basal layer of the epithelium were imaged. In accordance with Charman′s theoretical model [30], the posterior surface of the flap may have been larger than the ablated anterior surface of the stroma. Combined with severe flap edema on the first day, the flap probably twisted and compressed radially to match the two surgical wound surfaces. Among the five layers of cornea, only Bowman′s layer and the underlying stroma contain collagen fibrils that support corneal tensile strength [31]. During the corneal flap creation procedures, the integrity of the Bowman′s layer was broken. Thus, the microdistortions appeared after the flap was relocated. This phenomenon, captured by UHR-OCT, might be a response to the structural and reparative changes of the cornea. This study only followed the patients to 1 month. It might be the starting point to study the function of Bowman′s layer. Longitudinal study of long term follow-ups after refractive surgery would help to explain more related clinical problems.

Epithelial integrity plays a role in maintaining ocular surface health after refractive surgeries. The epithelium, which is composed of five to seven cell layers of approximately 50 to 52 μm thickness at the center of the cornea, is self-renewing and highly active [3,16,17,21,22]. The epithelial profile is dynamically stable over time in healthy corneas, thus maintaining the refractive power. There are several studies that focused on epithelial thickness changes after LASIK. Using time-domain OCT of 10 μm axial resolution, Wang et al. reported no central epithelial thickness changes 1 day after surgery [17]. However, after 1 month, the thickness of the epithelium was significantly increased. Using very high-frequency digital ultrasound, Reinstein et al. reported central epithelial thickening of 22%, 58%, and 20% within 1 day, from 1 day to 1 month, and between 1 and 3 months after LASIK [16]. Non-uniform lenticular epithelial thickening, as previously reported by Reinstein et al., occurred and probably correlated with myopic shift [15]. However, other studies reported that the central epithelial thickness was stable at 1 week [32] and 1 month [14] after LASIK surgery.

The UHR-OCT used in this study had a resolution of 3 μm in corneal tissue, thus achieving highly precise and accurate measurements. We found that the thickness of the central epithelium increased by about 3 μm at 1 day after Moria and FEMTO flap creations. Between 1 day and 1 week, the epithelial thicknesses decreased significantly and then increased again up to 1 month after SBK. The finding of increased thickness at 1 month is consistent with previous studies [14–17]. Epithelial hyperplasia during wound healing might contribute to this change. The changes in epithelial thickness might be the result of immediate epithelial remodeling in response to curvature changes [16] due to both the irregular stromal surface and Bowman′s layer microdistortions [15,16].

Flap thickness is impacted by different refractive surgery procedures and devices. During LASIK, systemic and/or random errors that cause large deviation in the actual flap thickness from the nominal setting may increase the risk for keratectasia [33]. Previous biomechanical studies showed evidence that the anterior third stroma provided the most cohesive tensile strength for entire cornea [34,35]. Dupps et al. reported that only Bowman′s layer and the stroma contain collagen fibrils that are responsible for the majority of the biomechanical tension of the cornea [31]. Thus creating a thin flap with a uniform profile may reduce the risk of corneal ectasia after ablation. Durrie et al. reported that the biomechanical impact on the cornea of SBK was similar to PRK, which in that study, was performed on the contralateral eye [36]. However, visual recovery, including uncorrected visual acuity, best-spectacle corrected vision, high order aberration, and contrast sensitivity function, for both LASIK and SBK patients was quicker than that for PRK patients.

In the current study, flap thickness measured by UHR-OCT was 96 μm for the Moria group and 106 μm for the FEMTO group at 1 day after SBK, and the changes during the one month follow-up were similar for both groups. At 1 month, flap thickness of both groups increased by 2 μm compared to the thickness at 2 weeks. The variations in SBK flap thickness were similar to those reported for LASIK in previous studies. With a time domain OCT prototype, Li et al. reported that flap thickness of an IntraLase group decreased nearly 3 μm between 1 day and 1 week after surgery [18]. Wang et al., using a custom-made 1310-nm wavelength OCT system, reported that flap thickness increased 3 μm from 1 week to 1 month after LASIK [17].

Different methods for flap creation can result in variations in flap and stromal bed structure. According to several published papers, ultra-thin flaps of 90 μm increase the inflammatory response [37], rainbow glare [38], laser tracker failure [39] and interface haze [40]. In addition, menisci created with a microkeratome and having a smooth interface between flap and stromal bed can result in interface slippage and delayed wound healing [41]. Further, femtosecond lasers create uniform flaps having microphotodisruptions that increased the adhesion between interfaces [11,41]. Considering all of these factors, long term clinical effects of SBK with different flap creation methods and thickness settings need to be studied further.

For total corneal thickness, the UHR-OCT system recorded significant decreases of about 9 μm from 1 day to 1 week after surgery for both the Moria and FEMTO groups. However, from 1 day to 1 month, the total corneal thickness did not increase significantly. The phenomenon of changes in corneal thickness was also observed in previous studies. Durairaj et al. reported cornea edema was greatest after repositioning the flaps, and it decreased slightly at the first day [42]. Edema continued to decrease until the fifth day after the surgery and then remained stable. Nagy et al. used ultrasound to obtain corneal thicknesses of 501.6 μm on the first day, 487.4 μm on the fifth day, and 481.8 μm at 1 month post-operation [43]. However, with the Humphery OCT system (Zeiss Humphrey, San Leandro, CA, USA), Maldonado et al. reported corneal thickness increased 6.6 μm at 1 month compared to 1 day post-surgery [44]. All of these reports validate the occurrence of total corneal thickness variations after refractive surgery. The correlation of visual quality problems with alterations in the thickness map of the entire cornea is a topic that warrants further study in the future.

This is the first use of UHR-OCT images to show all of the corneal sublayer thickness changes after SBK surgery. We documented the presence of post-operative changes in the thickness of the central epithelium and Bowman′s layer, but not of the peripheral corneal sublayers. More clinically meaningful results will be obtained if thickness maps of the central and peripheral corneal layers can be calculated and reconstructed in future studies. Our follow-up period to monitor the early corneal responses to SBK was limited to one month. Long-term observation with a large sample size and with more clinical instruments, such a confocal microscope and an ocular response analyzer, may offer more detailed information to evaluate the effect and efficiency of SBK surgery.

In summary, the central Bowman′s layer thickness increased 1 day after SBK. Flap creation methods of Moria microkeratome and femtosecond laser did not have significantly different impacts on the thickness of Bowman′s layer.

## Supporting Information

S1 Checklist (DOC)

S1 Protocol (DOC)

S1 Ethics Approval (DOCX)
