# Supplementary material for: The Impact of Flap Creation Methods for Sub-Bowman’s Keratomileusis (SBK) on the Central Thickness of Bowman’s Layer
Source: PLoS One. 2015 May 4;10(5):e0124996. doi: 10.1371/journal.pone.0124996 (PMC4418749; doi:10.1371/journal.pone.0124996)
Supplement: S1 Protocol — (DOC) [file pone.0124996.s002.doc]

**Wenzhou Medical University OCT Lab**


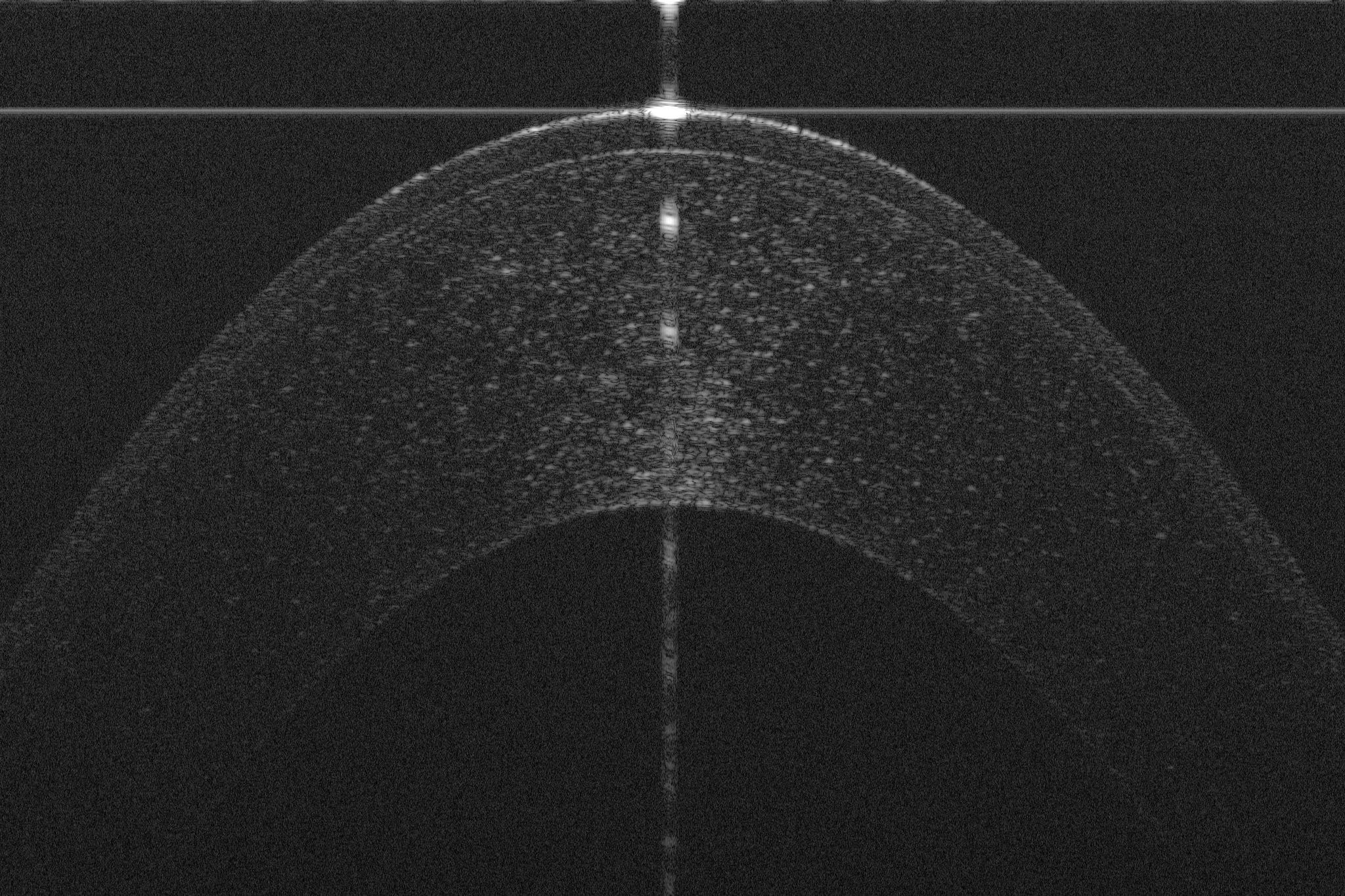


Optical Coherence Tomography 3D Cornea Reconstruction and Clinical Applications

Contents

1. Study instructions……………………………………………..………...……… 3
2. Study methods ……………………………………..…………….……………5
3. Study design ……..………………………………..…………………………… 6
4. Subjects information…………………………………………………………… 13
5. Study records ……………………………..…………………………………… 14
6. References……………………………………………………………………… 15
7. Attachments …………………………………………………………………… 17

Naming rules

Consent form

## 1 Study instructions

- 1. **Backgrounds**

The excimer laser served as a surgical tool was first introduced into ophthalmology and refractive surgery almost 30 years ago. Compared with photorefractive keratectomy (PRK), laser situ keratomileusis (LASIK）reduce postoperative pain, haze and accelerate visual recovery. LASIK is nowadays the most popular corneal refractive surgery around world. With the development of femtosecond laser (FEMTO), flap creation methods were improved at the same time. Sub-Bowman’s keratomileusis (SBK) combines the advantages of PRK and LASIK, creates thinner flaps in accurate and precise ways. Thus, SBK improved the efficacy, tolerability, and safety of refractive surgery.

Keratoconus is a bilateral asymmetry ectasia disease, which is characteristic with cornea thinning. However, when patients were in early stage, there were no obvious clinical signs and symptoms. Even the most sensitive corneal topography could not diagnosis. The only complain was reduced visual acuity. If the preoperative screening missed such kind of patients, cornea refractive surgery might lead to keratectasia. Thus, it is important to diagnose these subclinical patients in the stage of cornea thickness thinning.

Optical coherence tomograghy (OCT) relays on the principles of low-coherence interferometry to differentiate corneal sublayer structures. Anterior segment OCT advantaged in high acquisition speed, high axial resolution and non-invasive contact. With improved imaging technologies and surgical skills, it is promising that the diagnosis and treatment method will benefit more patients and scientific researchers.

**1.2 Aims**

A. Using OCT to obtain corneal images before LASIK, post LASIK 1 day, 1 week, 2 weeks, 1 month. Compare the differences between two flap creation methods.

B. Observe the cornea images acquired from keratoconus patients in different stages. Analysis thickness data and the relationship between the thickness and stage.

**1.3** **Hypothesis**

During the observation of following-ups, OCT images might capture the changes before and after LASIK. The relationship between flap creation methods, ablation depth and corneal thickness might be captured. After LASIK, cornea edema might appear on the OCT images. As the longer observation time after surgery, cornea edema might reduce. Slit lamp will be used during following-ups and assist to image cornea. OCT will image the interfaces and boundaries of corneal sublayers and flaps.

At the same time, clinical exam results will be documented. Combined with OCT images, correlation between refractive regression, corneal curvature and corneal edema will be studied.

Keratoconus patients will be examed by clinical instruments. Patients of different stages will be included. OCT images will analysis combined with clinical data to study the relationship between the corneal thickness and disease courses.

**2 Study methods**

**2.1 Ultra-long scan depth OCT (UL-OCT)**

Custom-built OCT equippmented with superluminescent diode light source (SLD) with a wavelength centered at 830 nm and band width of 50 nm. The output power is less than 1.30 mW as safety limit (ANSI Z136.1). The scan depth is 7.8 mm in the air with scan speed of 24000 A-line/sec. The axial resolution was ~6.0μm and lateral resolution was ~15μm. This instrument capture images at horizontal and vertical directions. Real-time image in two directions might help to relocation on the corneal vertex. OCT probe was fixed on the slit lamp and easy to adjust the imaging capture positions.

UL-OCT is used to obtain 3 dimensional corneal images for corneal thickness.

**2.2 Ultra-high resolution OCT (UHR-OCT)**

Custom-built UHR-OCT equippmented with superluminescent diode light source (SLD) with a wavelength centered at 840 nm and band width of 100 nm. The output power is less than 1.30 mW as safety limit (ANSI Z136.1). The scan depth is 2.0 mm in the air with scan speed of 24000 A-line/sec. The axial resolution was ~3.0μm and lateral resolution was ~15μm. This instrument capture images at horizontal and vertical directions. Real-time image in two directions might help to relocation on the corneal vertex. OCT probe was fixed on the slit lamp and easy to adjust the imaging capture positions.

UHR-OCT is used to obtain 3 dimensional corneal images for corneal epithelium, Bowman’s layer and corneal flaps.

**2.3 Lab conditions**：dim light, temperature 1.5-25^o^C，humidity 30-50%.

**2.4 Clinical exams**

(1) auto-refractor

(2) non-contact tonometer

(3) phoropter

(4) corneal topography（Pentacam, Toplyzer）

(5) Wavefront sensor (iTrace)

(6) Visante-OCT

(7) slit-lamp microscope

(8) A-scan ultrasound pachymetry

**3 Study design**

**3.1 Subjects：**recruit subjects as protocol.

**3.2.1 Inclusion criteria**

Normal group：

1. no eye surgery,trauma; 2. no other eye dieases: glaucoma,retina disease; 3. no limiting image quality diseases: severe dry eye, eyelid calculi, conjunctivitis and pterygium; 4. quit contact lens: RGP > 4 weeks, SCL > 2 week; 5. no fixing problem.

Refractive surgery group：

1. age：≥18yrs；2. refractive status：sphere：-1.00~-12.00 D, cylinder：≤ -6.00 D；3. refractive status maintain stable in recent 2 yrs，progressive ≤ 0.50 D per year.

Keratoconus group：

Suspected and diagnosed Keratoconus patients ：1. history of myopia and astigmatism；2. corrected visual acuity <1.0；3. abnormal corneal topography：central anterior corneal curvature >47D, curvature difference of inferior 3mm and superior 3mm of anterior cornea IS>3D, binocular curvature difference >1D；4.slit lamp observe one of the signs：corneal stromal thinning, corneal cone, Fleischer’s ring, Vogt stria, cornea scar.

**3.2.2 Exclusion criteria**

1. eye conditions not available; 2. active inflammation; 3.retina dieases; 4. system disease affect eyes; 5. other disease affect wound healing; 6. carrer limiting.

**3.3 Study procedures**

**3.3.1 Clinical screening**

Normal group：

Clinical exams：uncorrected visual acuity, computer optometry, non-contact tonometer, subjective refraction, corneal topography (Pentacam, Toplyzer), pupil size, slit lamp imaging, Visante-OCT, mydriasis test, A-scan ultrasound pachymetry, axial lengths, lacrimal passage check.

Slit lamp imaging：capture central cornea images with diffused light, narrow band light, wide band light.

Refractive surgery group：

Pre-operation exams：uncorrected visual acuity, computer optometry, non-contact tonometer, subjective refraction, corneal topography (Pentacam, Toplyzer), pupil size, slit lamp imaging, Visante-OCT, mydriasis test, A-scan ultrasound pachymetry, axial lengths, lacrimal passage check.

Slit lamp imaging：capture central cornea images with diffused light, narrow band light, wide band light.

Keratoconus group：

Clinical exams：uncorrected visual acuity, computer optometry, subjective refraction, corneal topography (Pentacam, Toplyzer), slit lamp imaging, Visante-OCT, mydriasis test, A-scan ultrasound pachymetry, axial lengths, lacrimal passage check.

Slit lamp imaging：capture central cornea images with diffused light, narrow band light, wide band light.

**3.3.2 Formal study**

**（1）visiting spots：**Pre, post 1 day, 1week, 2 weeks, 1month, 3months.

**（2）OCT scanning：**according to random number table, select one eye.

**A. UL-OCT：**run: C:\OCT\combined_x-y\OCT-T34-Combined_1.vi

1.Obtain OCT images as following：

**1）Central 3D radial scanning：**calibrated scan width 18.291mm.Scanning setting：“X/Y scan width” set as 15, “line rate” set as 24000 k/A-line，“scan pattern” set as 2048×324 mm radial.


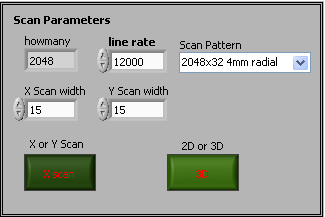


**2）Horizontal 2D scanning：**calibrated scan width 12.139 mm. Scanning setting：“X/Y scan width” set as 10, “line rate” set as 24000 k/A-line，“scan pattern” set as 2048×324 mm.


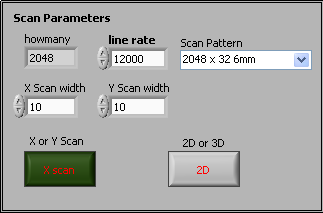


**3）Vertical 2D scanning：**calibrated scan width 12. 248 mm. Scanning setting：“X/Y scan width” set as 10, “line rate” set as 24000 k/A-line，“scan pattern” set as 2048×324 mm.


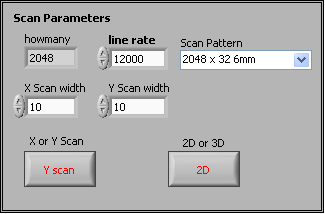


**2. Naming rules：** NO1_XX_S_1M_OD_2D_X10_1

**3. Data saving：** E:\LASIK_OCT\UL\Time\Subject

**4. Notice：**

(1) Using apex to adjust cornea vertex registration and relocation;

(2) Keep iris plane balanced;

(3) Check the image quality after each acquisition. Reacquire if the image quality is not good enough.

(4) Open the eyelid wide to avoid eye lash artifacts.

(5) Check 3D scanning image quality. Require if the reconstruction is not smooth.

**B． UHR-OCT：**run: C:\OCT\combined_x-y\OCT-T34-Combined_1.vi

1. Obtain OCT images as following：

**1）Central horizontal 2D scanning：**calibrated scan width 8.425 mm. Scanning setting：“X/Y scan width” set as 7, “line rate” set as 24000 k/A-line，“scan pattern” set as 2048×326 mm.


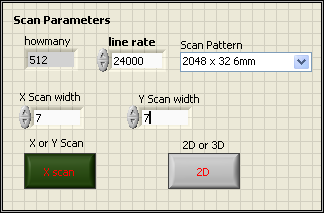


**2）Nasal horizontal 2D scanning：**calibrated scan width 8.425 mm. Scanning setting as “1）Central horizontal 2D scanning”.

**3）Temporal horizontal 2D scanning:** calibrated scan width 8.425 mm. Scanning setting as “1）Central horizontal 2D scanning”.

**4）Central vertical 2D scanning：**calibrated scan width 8.418 mm. Scanning setting：“X/Y scan width” set as 7, “line rate” set as 24000 k/A-line，“scan pattern” set as 2048×326 mm.


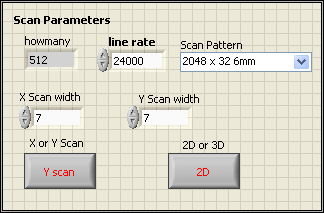


**5）Superior vertical 2D scanning：**calibrated scan width 8.418 mm. Scanning setting as “1）Central vertical 2D scanning”.

**6）Inferior vertical 2D scanning：**calibrated scan width 8.418 mm. Scanning setting as “1）Central vertical 2D scanning”.

**7）Central 3D radial scanning：**calibrated scan width 8.418 mm. Scanning setting：“X/Y scan width” set as 10.5, “line rate” set as 24000 k/A-line，“scan pattern” set as 2048×324 mm radial.


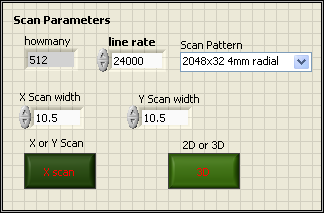


**2. Naming rules：** NO1_XX_S_1M_OD_N_2D_X10_1

**3. Data saving:**  E:\LASIK_OCT\UHR\Time\Subject

**4. Notice：**

(1) Using apex to adjust cornea vertex registration and relocation;

(2) Keep iris plane balanced;

(3) Check the image quality after each acquisition. Reacquire if the image quality is not good enough.

(4) Open the eyelid wide to avoid eye lash artifacts.

(5) Check 3D scanning image quality. Require if the reconstruction is not smooth.

**3.4 OCT imaging processing:**

UL-OCT: using custom developed automated software to reconstruct cornea.

UHR-OCT: using custom developed automated software to obtain thicknesses of each corneal sublayers.

Imaging processing: (1) raw images; (2) manual measurements (J-OCT): interfaces and boundaries of each sublayer; (3) automated Matlab code: detailed measurement results of all corneal layers.

**3.5 Post refractive surgery controls**

Flap creation methods：

1. One-Use Plus SBK Moria microkeratome (Moria, Antony, France);
2. IntraLase Femtosecond Laser (Abbott Medical Optics, Santa Ana, CA, USA).

Pre operation medication：

(1) Levofloxacin

(2) Artificial tears (one of three)

Sodium carboxymethylcellulose

Dextran hydroxypropyl methyl cellulose

Polyvinyl Alcohol

Post operation medication：

(1) Levofloxacin

(2) Artificial tears (one of three)

Sodium carboxymethylcellulose

Dextran hydroxypropyl methyl cellulose

Polyvinyl Alcohol

(3) Fluorometholone Acetate

Notice: Keep records if temporarily adjust the medication.

**3.6 Data analysis:**

All data were collect and save in Excel to build a database.

Statistical methods were used to analysis data.

1. T-test；
2. Liner Regression, Pearson’s correlation.

4 Subjects information

**4.1 Consent information**

This study was approved by the Office of Research Ethics, Wenzhou Medical University, Wenzhou, Zhejiang, China. Written consent form was obtained from each subject. Subjects were treated according to tenets of the Declaration of Helsinki. All subjects were recruited at the Refractive Surgery Division of the Affiliated Eye Hospital of Wenzhou Medical University.

Study was performed between 8:00 AM~5:00 PM. Totally no more than 0.5 hour.

**4.2 Treatment arragement**

If adverse effects happen during the study, all subjects will treated by ophthalmologist clinically.

**4.3 Adverse events**

Since this study is totally non-contact and non-invasive, the rate of adverse events was low. The possible adverse events were uncomfortable feeling caused by long time fixing and so on. All subjects will be treated by clinical ophthalmologist no matter the reasons for the uncomfortable. Relative doctors will get involved in the treatment when it necessary. Subjects can exit and quit the study when they are willing to. Adverse events might be kept as video records when necessary. Adverse events should be reported to the Office of Research Ethics and make detailed treatment plans.

**4.4 Casing study**According to tenets of the Declaration of Helsinki, subjects can exit the study anytime belonging to individual wishes. If the cooperation was not that good, study will be quited. All subjects will be treated by clinical ophthalmologist no matter the reasons for the uncomfortable. Subjects may rejoin the study when the issues were properly solved.

**5 Study records**

Each subject will be documented separately. Researcher should keep detailed and private information for each subject. The reports of each visiting will be maintained and concluded after each study time spot. Researcher should check the study results and sign the names and date with black pen formally. If the information need to be corrected, single horizontal line should be used to mark over the records and sign with the researcher’s name and date. The corrected part should be maintained recognizable. If the records are not able to be distinguish, “*” should be marked with commons. The OCT image results of each subject will be recorded in DVD discs and named after naming rules. The results may be used to compare with clinical results when necessary. All the records will be well kept at least 5 years.

**6 References**

1. 王勤美. 屈光手术学.北京：人民卫生出版社, 2011.

2. 陆豪, 李海生. 眼光学相干断层扫描成像术原理和临床应用.上海：上海兴界图书出版公司, 2008.

3. Jianhua Wang, MD, PhD; Joseph Thomas, MD; Ian Cox, PhD. Corneal Light Backscatter Measured by Optical Coherence Tomography After LASIK. *J Refract Surg.* 2006;22:604-610.

4. Yuehua Zhou, MD; Lei Tian, MD; NingLi Wang, MD; Paul J. Dougherty, MD. Anterior Segment Optical Coherence Tomography Measurement of LASIK Flaps: Femtosecond Laser vs Microkeratome. *J Refract Surg.* 2011;27(6):408-416.

5. Yan Li, MS,1 Raj Shekhar, PhD,2 David Huang, MD, PhD3. Corneal Pachymetry Mapping with High-speed Optical Coherence Tomography. *Ophthalmology* 2006;113:792–799.

6. Yan Li, MS,1 Marcelo V. Netto, MD,2,3 Raj Shekhar, PhD,4 Ronald R. Krueger, MD,2 David Huang, MD, PhD2. A Longitudinal Study of LASIK Flap and Stromal Thickness with High-speed Optical Coherence Tomography. *Ophthalmology* 2007;114:1124–1132.

7. Miguel J. Maldonado, MD, PhD, Lilian Ruiz-Oblitas, MD, Juan M. Munuera, MD, PhD, Daniel Aliseda, MD, PhD, Alfredo Garcı´a-Layana, MD, PhD, Javier Moreno-Montan˜e´s, MD, PhD. Optical Coherence Tomography Evaluation of the Corneal Cap and Stromal Bed Features after Laser In Situ Keratomileusis for High Myopia and Astigmatism. *Ophthalmology* 2000;107:81–88.

8. Wang J, Abou SM, Perez VL, et al. Ultra-high resolution optical coherence tomography for imaging the anterior segment of the eye[J]. Ophthalmic Surg Lasers Imaging, 2011, 42: 15-27.

9. Li Y, Meisler DM, Tang M, et al. Keratoconus diagnosis with optical coherence tomography pachymetry mapping[J]. Ophthalmology, 2008, 115: 2159-2166.

10.Li Y, Tang M, Zhang X, et al. Pachymetric mapping with Fourier-domain optical coherence tomography[J]. J Cataract Refract Surg, 2010, 36: 826-831.

10.Ortiz S, Siedlecki D, Grulkowski I, et al. Optical distortion correction in optical coherence tomography for quantitative ocular anterior segment by three- dimensional imaging[J]. Opt Express, 2010, 18: 2782-2796.

11.Ishibazawa A, Igarashi S, Hanada K, et al. Central corneal thickness measurements with Fourier-domain optical coherence tomography versus ultrasonic pachymetry and rotating Scheimpflug camera[J]. Cornea, 2011, 30: 615-619.

12. Shankar H, Pesudovs K. Reliability of peripheral corneal pachymetry with the Oculus Pentacam[J]. J Cataract Refract Surg, 2008, 34: 7.

**7 Attachments**

**7.1 Naming rules**

**7.1.1 UL-OCT naming rules:**


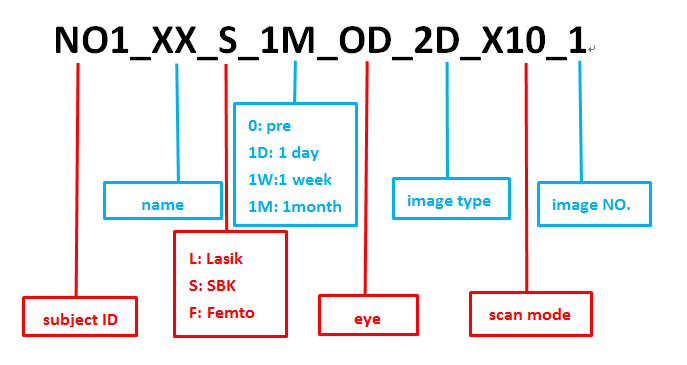

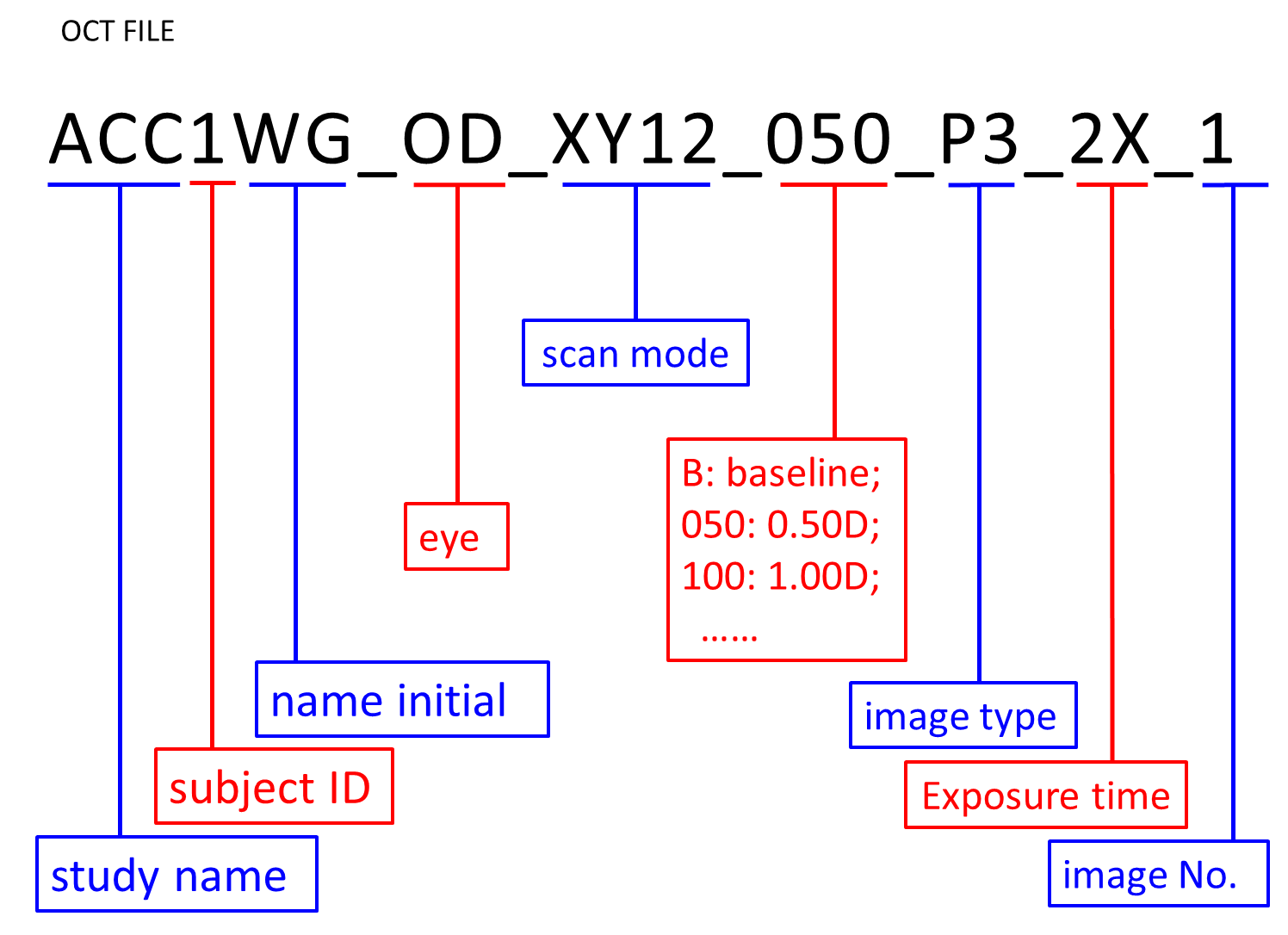


**7.1.2 UHR-OCT naming rules：**


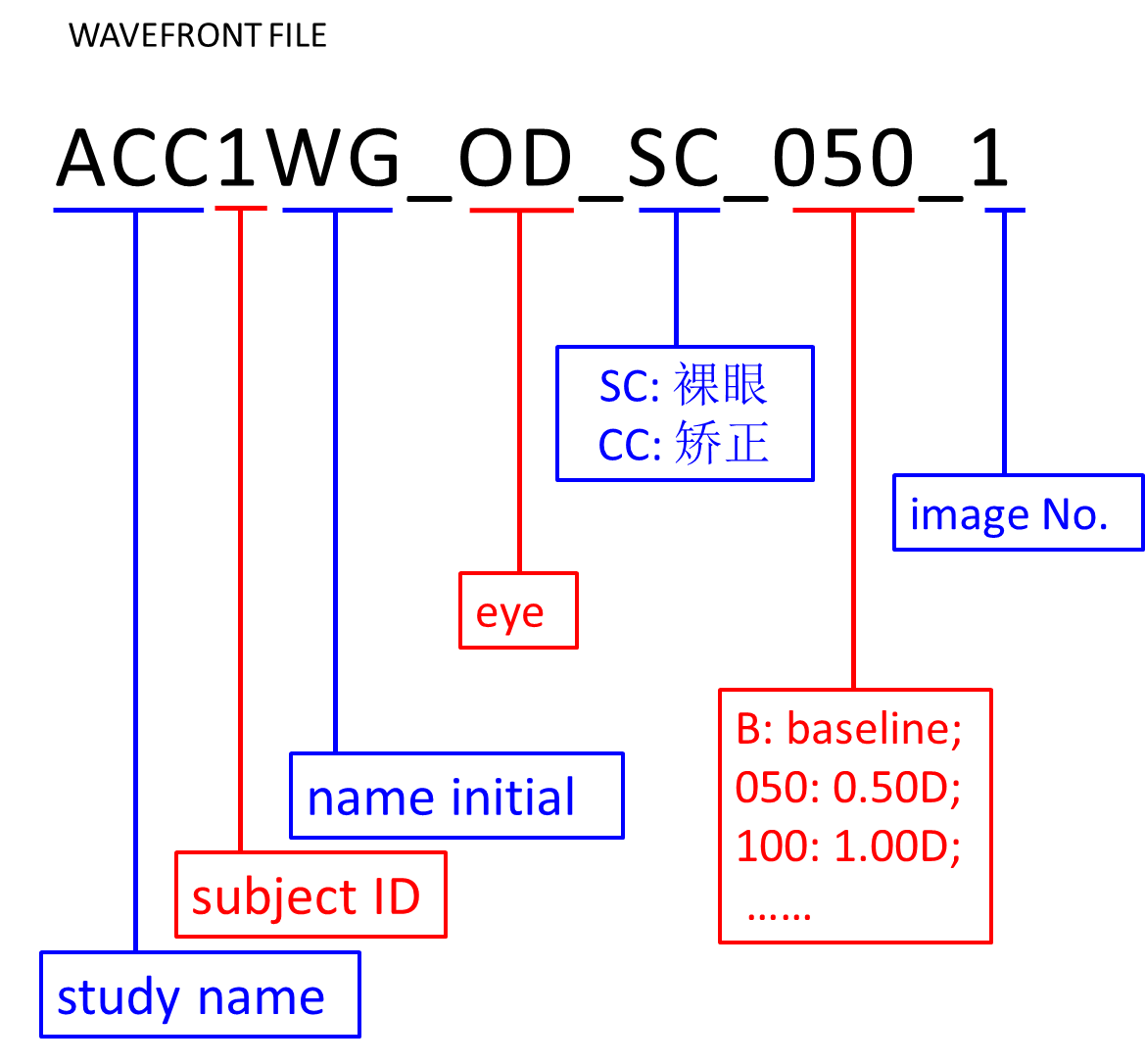


**
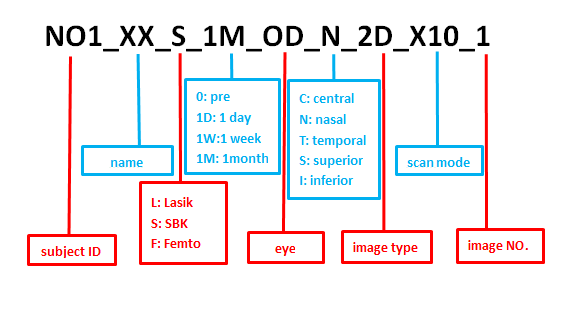
**

**7.2 Consent Form**

**Study title：**Optical Coherence Tomography 3D Cornea Reconstruction and Clinical Applications

**Study researchers：**Meixiao Shen, Zhe Xu, Liang Hu, Qi Chen, Jing Liu, Xiran Zhuang, Mei Peng, Lin Leng, Yilei Shao, Sisi Chen, Shenghai Huang, Jianghua Wang, Fan Lu *etc.*

**Instruction**

This consent form includes all the details about this study. You may read it carefully before participating in this study. You may ask any questions you cared about and seek answers from study researchers. We will patiently answer any questions. After that you can decide to join in the study or not.

**Study Aim**

This study will focus on the optical coherence tomography 3d cornea reconstruction and clinical applications.

**Study Procedures**

If you agreed to paticipate in this study, researcher will give you some clinical exams, like subjective refraction, Intraocular pressure measurement etc. After that, researches will image your cornea with OCT. The custom built OCT used in this study will not get direct contact on your cornea and bring any invasive effects. While examing, you just need to sit on the chair. Reseachers will ask you to adjust head positions and keep fixing on the visual target. The results from this study will be published in professional journals and used in other scientific studies. However, we will not disclose personal information of you privacy.

**Risks**

The exams will not lead to any medical risks have already known.

**Proprietary interests**

The researchers have no proprietary interest in any materials or methods described within this study.

**Costs**

You don’t need to pay for any exam in this study.

**Payments**

All the OCT image you taken is for free. You will not gain any payments from this study.

**Pravicy**

We promise the pravicy of you personal information. Study results will be published and communicated on the conferences and journals. However, we will not notice any your personal pravicy. We will gain your authorization and agreement if your healthy information and exam results are needed while publication. If you agree to participate in this study, we will keep your study records carefully. Some of these information may be used in other studies. We will use it belong to the tenets of the Declaration of Helsinki and study protocol. You will get a copy of this consent form. Your authorization will be valid till the end of the study. We will strictly keep your information private. You may cease or exit this study whenever you wish and stop your authorization. As your authorization cancelled, you will exit this study. And the payment will also be cancelled.

As a voluntary participation, you have the right to refuse to sign the authorization. It will not be a part of the study. If you sign the authorization, you will allow us to obtain healthy information of your eyes and use in the study.

**Voluntary participation**

You feel willing to participate in this study. You may stop the study any time you wish. After your quitting, all the information will still be kept privately.

**Research contacts**

If you want to know more details about this study, please contact with:

**Participants**

**I have already read the consent form and got informed about all the unknown questions. I agree to participate in this study.**

**Name：____________________ Signature：__________________**

**Tel：______________________ Date：__________________**

**Study researcher**

**I have already explain all the details of this study to the subject. I agree to give a copy of consent form to the subject.**

**Researcher：____________________ Signature：__________________**

**Date：__________________**
