# Supplementary material for: The Impact of Flap Creation Methods for Sub-Bowman’s Keratomileusis (SBK) on the Central Thickness of Bowman’s Layer
Source: PLoS One. 2015 May 4;10(5):e0124996. doi: 10.1371/journal.pone.0124996 (PMC4418749; doi:10.1371/journal.pone.0124996)
Supplement: S1 Ethics Approval — (DOCX) [file pone.0124996.s003.docx]

| **Ethics Approval** |
| --- |
|  |
| **No. KYK [2013] 4. School of Ophthalmology and Optometry** |
| **Project title:** The cornea structure reconstruction of three-dimensional optical coherence tomography (OCT) images and clinical applications |
|  |
| **Project field:** Ophthalmology and Optometry |
| **Lab manger:** Meixiao Shen, Fan Lu |
| This ethical approval form certificated the ethic feasibility of human research study of "The cornea structure reconstruction of three-dimensional optical coherence tomography (OCT) images and clinical applications". All the staffs, research assistants and research students obtained the ethic approvements of research with human subjects from the Office of Research Ethics, Wenzhou Medical University. |
|  |
|  |
|  |
|  |
|  |
|  |
|  |
|  |
|  |
|  |
|  |
|  |
|  |
|  |
|  |
|  |
|  |
| **Estimated date: Jan/15/2013** |
| **Office of Research Ethics** |
| **Wenzhou Medical University** |
